# Detecting genomic regions associated with a disease using variability functions and Adjusted Rand Index

**DOI:** 10.1186/1471-2105-12-S9-S9

**Published:** 2011-10-05

**Authors:** Dunarel Badescu, Alix Boc, Abdoulaye Baniré Diallo, Vladimir Makarenkov

**Affiliations:** 1 Département d'lnformatique, Université du Quebec a Montreal, C.P. 8888, Succursale Centre-Ville, Montreal (Quebec), H3C 3P8, Canada

## Abstract

**Background:**

The identification of functional regions contained in a given multiple sequence alignment constitutes one of the major challenges of comparative genomics. Several studies have focused on the identification of conserved regions and motifs. However, most of existing methods ignore the relationship between the functional genomic regions and the external evidence associated with the considered group of species (e.g., carcinogenicity of Human Papilloma Virus). In the past, we have proposed a method that takes into account the prior knowledge on an external evidence (e.g., carcinogenicity or invasivity of the considered organisms) and identifies genomic regions related to a specific disease.

**Results and conclusion:**

We present a new algorithm for detecting genomic regions that may be associated with a disease. Two new variability functions and a bipartition optimization procedure are described. We validate and weigh our results using the Adjusted Rand Index (ARI), and thus assess to what extent the selected regions are related to carcinogenicity, invasivity, or any other species classification, given as input. The predictive power of different hit region detection functions was assessed on synthetic and real data. Our simulation results suggest that there is no a single function that provides the best results in all practical situations (e.g., monophyletic or polyphyletic evolution, and positive or negative selection), and that at least three different functions might be useful. The proposed hit region identification functions that do not benefit from the prior knowledge (i.e., carcinogenicity or invasivity of the involved organisms) can provide equivalent results than the existing functions that take advantage of such a prior knowledge. Using the new algorithm, we examined the *Neisseria meningitidis* FrpB gene product for invasivity and immunologic activity, and human papilloma virus (HPV) E6 oncoprotein for carcinogenicity, and confirmed some well-known molecular features, including surface exposed loops for *N. meningitidis* and PDZ domain for HPV.

## Background

Many bacteria and viruses adapt to changing environmental conditions through several evolutionary mechanisms such as homologous recombination [1], nucleotide substitutions, insertions-deletions [2], horizontal gene transfer [3], etc. These mechanisms lead to the formation of different polymorphic strands of the same group of organisms, in which the variation on the DNA composition is spread randomly throughout the genomes. The survival of these strands depends on their ability to overcome the environmental changes [4]. One of the goals of comparative genomics consists of finding the variation among aligned genomic sequences in order to identify functional regions. Several comparative genomic tools allow the identification of genomic regions in an alignment that have evolutionnary patterns different from the neutral evolution. For instance, PhastCons [5] predicts, from a given alignment and the related phylogenetic tree, the genomic regions under negative selection. PAML [6-8] allows the comparison of synonymous versus non-synonymous mutations in an alignment in order to predict regions under selective pressure. RDP3 [9] and TOPAL [10] are software packages including several methods for detecting recombination. Most of these methods and software do not take into consideration external epidemiological evidence associated with many bacterial and virus strands. Such an evidence can allow the clustering of organisms based not only on the similarity of their genomic sequences, but also, on their association to different diseases. Hence, intra-specific and inter-specific variation among carcinogenic and non-carcinogenic human papilloma viruses can lead to the identification of regions related to carcinogenicity. In our previous works, we introduced a hit region identification function using prior knowledge information [11] and described the related validation framework based on Monte-Carlo simulations [12]. Then, we extended the latter study by presenting and testing four variants of the hit region identification function, still using the available prior knowledge [13]. In this paper, we present a new algorithm for the identification of specific genomic regions associated with an external disease. The introduced algorithm uses a bipartition optimization procedure to maximize a specific clustering function *Q*, based on inter- and intra-group variability, for each window position, over the given sequence alignment. It can be applied *with or without prior knowledge information* characterizing species in hand. Hit regions (i.e., putative regions related to a disease) can be validated using ARI [14] (a corrected-for-chance version of the Rand index [15]) and organismal bipartitions are constructed using the available epidemiological data. The new algorithm has been applied to two independent datasets: The human papilloma viruses and the *Neisseria meningitidis* data. The obtained results suggest that genomic regions with important biological features in both datasets can be associated with either carcinogenicity or invasivity.

## Dataset description

### Neisseria meningitidis dataset

*Neisseria meningitidis* is a Gram negative bacterium responsible for meningitis and septicemia. It has a relatively small genome size of 2.2 Mbp. In March 2011, the PubMLST database listed a total of 8,793 genetically distinct members of Neisseria organisms [16]. All these facts make *N. meningitidis* well suited for testing comparative genomics methods [17]. Proteins expressed under iron limitation (e.g. FrpB(FetA)) are considered as potential vaccine components [18]. Bacteria grown under iron starvation express several proteins, the most abundant of them being FrpB, a 70kDa outer membrane protein (OMP). It is expressed in large amounts in all strains, and antibodies against this protein appear to be bactericidal. A putative FrpB topology was first proposed with a 26-stranded β-barrel [19], and later reassessed to a plug domain and a 22-stranded *β*-barrel with 11 surface-exposed loops [20]. These loops are accessible to the host immune system, which produces natural antibodies against these regions. In general, bacteria express genetic sequence variability in order to evade this defense mechanism.

The data we considered, were classified on the invasivity basis using a list of identified hyperinvasive meningococci [21]. We then built a list of unique FetA sequence tags carried by the alleles of these organisms. Using local BLAST operations [22], we searched for the presence of these tags in the distinct sequences belonging to the selected multiple sequence alignment (MSA), first examined in [13]. We classified as belonging to the invasive category (subset *X*) any allele that contained at least one of the selected invasive tags. All the other alleles were put in the non-invasive category (subset *Y*)*.* We annotated the MSA with the information regarding surface-exposed loops, beta-sheets and periplasmic loops [20]. Translating indexes from the amino-acid sequences to DNA sequences were also computed. Each single value of the hit region identification function *Q* (the *Q*-type functions will be used to identify genetic regions that may be related to a disease) corresponds to an interval of a certain length (i.e., 9 or 20 nucleotides in this study) and depends on the starting position of the sliding window used in our algorithm.

### Human papilloma virus dataset

Human papilloma viruses (HPV) have a causal role in cervical cancer with almost half a million new cases occurring each year [23-25]. About a hundred of HPV types have been identified, and the whole genomes of more than eighty of them have been sequenced (see the latest Universal Virus Database report by International Committee on Taxonomy of Viruses (ICTV)). A typical HPV genome is a double-stranded, circular DNA genome of size close to 8 Kbp, with a small set of genes (L1, L2, E1, E2, E4, E5, E6 and E7). In this study, we focused on the gene E6, which is predominantly linked to cancer due to the binding of its product to the p53 tumor suppressor protein. It contains a PDZ domain-binding motif (-X-T-X-V) at its carboxy terminus, which is essential for targeting the PDZ proteins for proteasomal degradation. Such proteins include hDlg, hScrib, MAGI-1, MAGI-2, MAGI-3 and MUPP1 [26]. The interaction between E6 and hDlg, or the other PDZ domain-containing proteins, may be an underlying mechanism in the development of HPV-associated cancers [27]. The gene E6 was also shown to contain two stable folded domains, E6N and E6C [28,29]. Models of these domains have been built in the absence of complete crystallographic data [30].

To define carcinogenic types, we used the epidemiological data from a large international survey on HPV in cervical cancer and from a multicenter case-control study conducted on 3,607 women with histologically confirmed cervical cancer [31,32]. More than 89% of them had squamous cell carcinoma (i.e., Squam cancer) and about 5% had adenosquamous carcinoma (i.e., Adeno cancer). More than a half of the infection cases were due to the types 16 and 18 of HPV, which are later referred to as High-Risk HPV [33]. In this study, we examined the content of the gene E6 for 83 different HPV types.

We fixed the window size to 20 nucleotides for HPV datasets in order to be consistent with our previous works [11,12], where we conducted simulations with windows of different sizes and used the size of 20 bp to present the results. In the same way, we considered the window size of 9 nucleotides for the *N. meningitidis* dataset to be consistent with another our study [13].

## Methods

### Description of the algorithm

**Figure 1:**
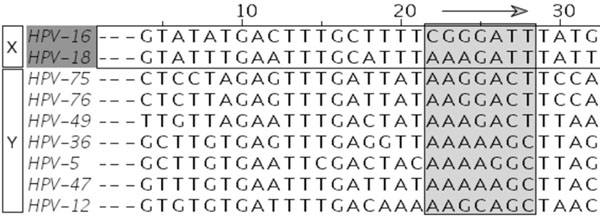
**Sliding window procedure** Sliding window of a fixed width was used to scan the HPV gene E6. The sequences in black belong to the set *X* (carcinogenic HPV; in this example HPV 16 and 18), all the other sequences belong to the set *Y* (non-carcinogenic HPV). The HPV type is indicated in the left column.

The new algorithm takes as input a MSA established for a set of organisms. Assume that this set of organisms is partitioned into two different subsets according to a boolean criterion (e.g., invasivity vs. non-invasivity or carcinogenicity vs. non-carcinogenicity). The corresponding subsets are denoted *X* (invasive/carcinogenic) and *Y* (non-invasive/non-carcinogenic), respectively. The region of interest is scanned using a non-overlapping sliding window, as shown in Figure 1, of a fixed width (20 sites for HPV and 9 sites for *N. meningitidis*)*.* For each window position, we carry out a bipartition optimization algorithm in order to search for maximum values of the hit region identification function. A specific version of the *Q*-type function (see below) can be taken as the algorithm parameter. We denote by *Q*′ a specific version of the *Q*-type function computed under condition that the subsets bipartition is unknown (i.e., prior knowledge). The complete algorithmic scheme is presented in Algorithm 1 in Additional file 1 .

#### Clustering using the Q-type functions

To perform the clustering of our data into two groups *A* and *B*, we first calculate the intragroup variability of the sequences from the group *A*, denoted by *V*(*A*), the group *B*, denoted by *V*(*B*), and, finally, the intergroup variability *D*(*A*, *B*), as described in Equations 1, 2 and 3. These measures are defined as the means of the squared Hamming distances, *dist*, among the sequence fragments (bounded by the sliding window position) of the taxa from the group *A* only, from the group *B* only, and between the sequence fragments from the distinct groups *A* and *B:*(1)(2)(3)

In [11-13] four different hit region identification functions, *Q*_1_, *Q*_2_, *Q*_3_ and *Q*_4_, which could be summarized by the following equation, were defined:(4)

where the [*k*, *l*] combinations are as follows: .

The function *Q*_4_ (Equation 5), along with new versions of the hit region identification function, denoted by *Q*_5_ (Equation 6) and *Q*_6_ (Equation 7), will be tested and discussed in this study:(5)(6)(7)

#### Measuring the agreement between the reference and the optimal calculated bipartitions using the Adjusted Rand Index (ARI)

The Adjusted Rand Index [14] has become a criterion of choice for measuring agreement between two partitions in clustering analysis [34]. Having a calculated bipartition *U*″ = *A|B* and a reference bipartition *U*′ = *X|Y*, for all  pairs of elements, one can compute how many of them fall into the same group and how many in different groups. One can then calculate ARI [35] according to Equation 8. ARI is the corrected-for-chance version of the Rand index [15]. It ranges between -1 and 1, and expresses the level of concordance between two bipartitions [14]. The values of ARI close to 1 indicate an almost perfect concordance between the two compared bipartitions, whereas the values close to -1 indicate a complete discordance between them:(8)

where , *a* is the number of pairs that are in the same group in the bipartitions *U*″ and *U*′, *b* is the number of pairs that are in the same group in the bipartition *U*″ and in different groups in the bipartition *U*′, *c* is the number of pairs that are in different groups in the bipartition *U*″ and in the same group in *U*′, and *d* is the number of pairs that are in different groups in the bipartitions *U*″ and *U*′*.*

#### Validation of the obtained hit regions using the Adjusted Rand Index

We define a new function *Q*″ reflecting the quality of the reference bipartition, as follows:(9)

The difference between *Q*′ and *Q*″ indicates the level of concordance of the reference bipartition *U*′ with the selected function *Q.* Throughout the paper, *Q* will denote the hit identification function using prior knowledge information, *Q*′ *–* not using any prior knowledge information and *Q*″ *–* using prior knowledge information and based on ARI.

#### Bipartition optimization

For each window position, we generated a fixed number of random initial bipartitions. For each such a bipartition, we moved elements from one subset to the other and back again in cycles, each time accepting the move that maximized the objective function *Q*, until no further improvement was possible. Once a local maxima was reached, we compared it to the best current value obtained for all starting random bipartitions tested so far. ARI was used to compare the level of concordance of the obtained bipartition (i.e., the one that was maximizing the given function *Q*) with the reference bipartition (carcinogenic vs. non-carcinogenic taxa for HPV and invasive vs. non-invasive taxa for *N. meningitidis*) given as a parameter to the algorithm.

#### Time complexity

The time complexity of the new algorithm carried out with an overlapping sliding window of a fixed width, and advancing one alignment site by step, is *O*(*l* × *n*^2^ × *w* × *r*), where *l* is the length of the MSA, *n* the number of considered species, *r* the number of random initial partition generations and *w* the window width. In order to ensure this complexity, we have to limit the optimization cycle to a constant number of iterations.

### Simulation study

In order to validate the hit region identification functions ,  and , we conducted a Monte-Carlo simulation study involving two major evolutionary mechanisms: Positive selection (PS) and Lineage specific selection (LSS). Two cases of group selection were also tested: The cases of the monophyletic and polyphyletic clustering. An approach involving the computation of p-values was implemented to asses the predictive ability of each of the three functions for each combination of evolutionary parameters. The following procedure was carried out. A phylogenetic tree *T* with 16 leaves was first generated using the algorithm described by Kuhner and Felsenstein [36]. The edge lengths of *T* were generated using an exponential distribution. Following the approach of Guindon and Gascuel [37], we added some noise to the tree edges in order to provide a deviation from the molecular clock hypothesis. The random trees yielded by this procedure had depth of *O*(*log*(16))*.* The tree was then rooted by midpoint. For the monophyletic test, the left and right sub-trees, denoted by *T*_1_ and *T*_2_, were determined, depending on the position of the root. For the polyphyletic test, two sets of leaves were randomly chosen and the corresponding sub-trees, denoted by *T*_3_ and *T*_4_, were extracted.

In the PS simulations, we used the original lengths of the edges of the subtrees *T*_1_ and *T*_2_ (i.e., monophyletic case), and *T*_3_ and *T*_4_ (i.e., polyphyletic case), while all edge lengths of *T* were gradually multiplied by the scaling factor *a*, varying from 0.05 to 1 (with the step of 0.05).

In the LSS simulations, all edge lengths of *T* were multiplied by 0.5 (thus simulating neutral evolution), while all edge lengths of *T*_1_ and *T*_3_ were multiplied by the scaling factor *a*_1_ = 0.5 + 0.025*x*, and all edge lengths of *T*_2_ and *T*_4_ by *a*_2_ = 0.5 – 0.025*x*, where *x* was varying from 1 to 19.

Second, we executed the SeqGen program [38] to generate random MSAs of nucleotide sequences along the edges of the phylogenetic trees constructed at the first step. The SeqGen program was used with the Jukes-Cantor model of sequence evolution [39]. DNA sequences with 440 bp were generated for each tree *T*. In addition, MSAs of the length 20 bp were generated for each of the trees *T*_1_, *T*_2_, *T*_3_ and *T*_4_. Two different variants of MSA were produced to simulate monophyletic and polyphyletic evolution. In the sequence alignment generated for the original tree *T*, we inserted those generated for the trees *T*_1_ and *T*_2_ in the monophyletic case, and those generated for the trees *T*_3_ and *T*_4_ in the polyphyletic case. The location of the inserted sequence blocks was known.

Thus, depending on the scaling factor parameters, for the PS case we simulated a variable homogeneous region inside a conserved context, and for the LSS case a more divergent region inside a neutral context. Third, we scanned the resulting sequence alignment using a sliding window of size 20 bp with the step of 1. We calculated the value of the hit region identification functions ,  and  for each fixed position of the window and assessed the proportion of their values that were higher than the reference value corresponding to the inserted region.

These steps were repeated over 100 different replicates and the distributions of the best (in each case) function over each combination of testing parameters were represented using quartiles.

## Results and discussion

**Figure 2:**
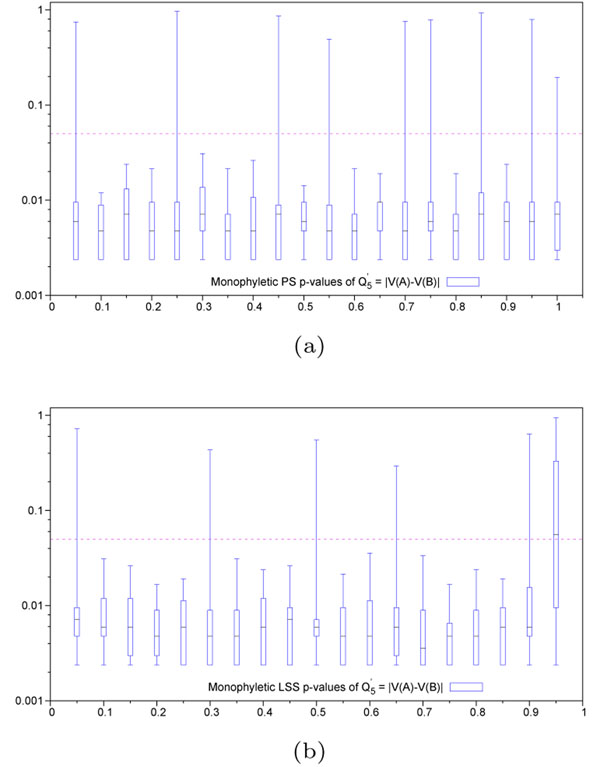
**p-values obtained for monophyletic evolution hit region detection** (a) Positive selection - Variable hit region inside conserved context. Quartile distribution of p-values obtained for the function . Abscissa represents scaling factor of the conserved context in which the variable hit region resides. Values close to 0 represent conservation (maximum discrimination), while values close to 1 represent variability (identical to context). Variable hit region is always maintained at a scaling factor of 1. Ordinate represents p-values in log-scale. Horizontal dashed line represents the significance threshold of 0.05. (b) Lineage specific selection - Heterogeneous hit region inside neutral context. Quartile distribution of p-values obtained for the function . Abscissa represents the difference in scaling factors among the two lineages present in the hit region. Values close to 0 represent homogeneous evolutionnary speed (similar to the neutral context in which it resides), while values close to 1 represent divergence among these lineages. Context is always maintained at a scaling factor of 0.5, simulating neutral evolution. Horizontal dashed line represents the significance threshold of 0.05. In the case of lineage specific selection, the value of the *Q*′*-tγpe* functions corresponding to 1 on the abscissa scale cannot be computed because it involves a sub-tree with 0 edge lengths.

**Figure 3:**
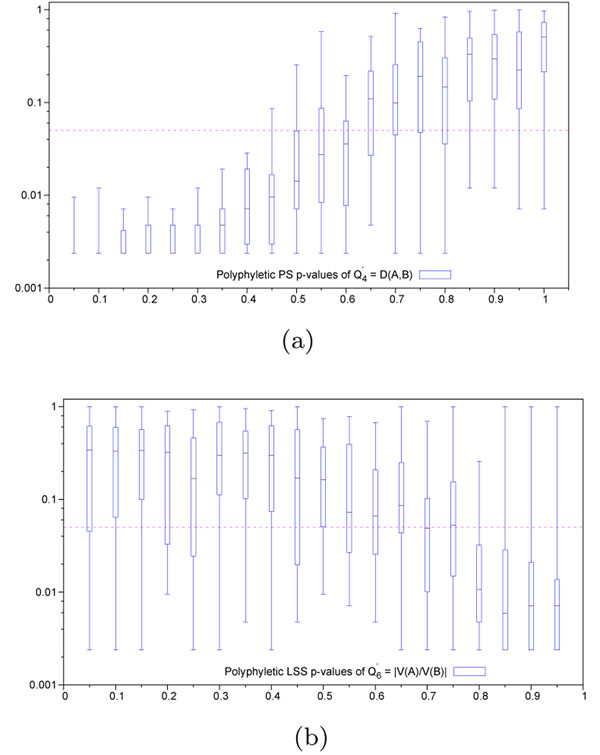
**p-values obtained for polyphyletic evolution hit region detection** (a) Positive selection - Variable hit region inside conserved context. Quartile distribution of p-values obtained for the function . Variable hit region is always maintained at a scaling factor of 1. Abscissa represents scaling factor of the conserved context in which the variable hit region resides. Values close to 0 represent conservation (maximum discrimination), while values close to 1 represent variability (identical to context). Ordinate represents p-values in log-scale. Horizontal dashed line represents the significance threshold of 0.05. (b) Lineage specific selection - Heterogeneous hit region inside neutral context. Quartile distribution of p-values obtained for the function . Context is always maintained at a scaling factor of 0.5, simulating neutral evolution. Abscissa represents difference in scaling factors among the two lineages present in the hit region. Values close to 0 represent homogeneous evolutionnary speed (similar to the neutral context in which it resides), while values close to 1 represent divergence among these lineages, and from the neutral context. Horizontal dashed line represents significance threshold of 0.05.

We proposed a new algorithm for finding genomic regions that may be related to a disease along with two new hit region identification functions *Q*_5_ and *Q*_6_*.* Both new functions along with the best existing function *Q*_4_ were tested in simulations. The functions yielding the best results for each case were illustrated in Figure 2: Monophyletic evolution (case a: PS, case b: LSS) and Figure 3: Polyphyletic evolution (case a: PS, case b: LSS). The remaining results for the ,  and  functions are presented in Additional file 1. Figures 2 and 3 clearly show that the hit zone identification in the monophyletic case is much easier than in polyphyletic case. We can suggest that in order to be recognized, the hit region has to have a different evolutionary speed than the context in which it resides. The polyphyletic lineage specific case represents the hardest evolutionary situation. Also, one can notice that different *Q*-type functions, ,  or , should be used in different practical situations.

The procedure for the identification of hit regions was carried out to detect the variability zones in the FrpB gene of *N. meningitidis* as well as the regions potentially responsible for cancer in the gene E6 of HPV. In both cases, we also carried out the ARI validation.

### Neisseria meningitidis analysis

**Figure 4:**
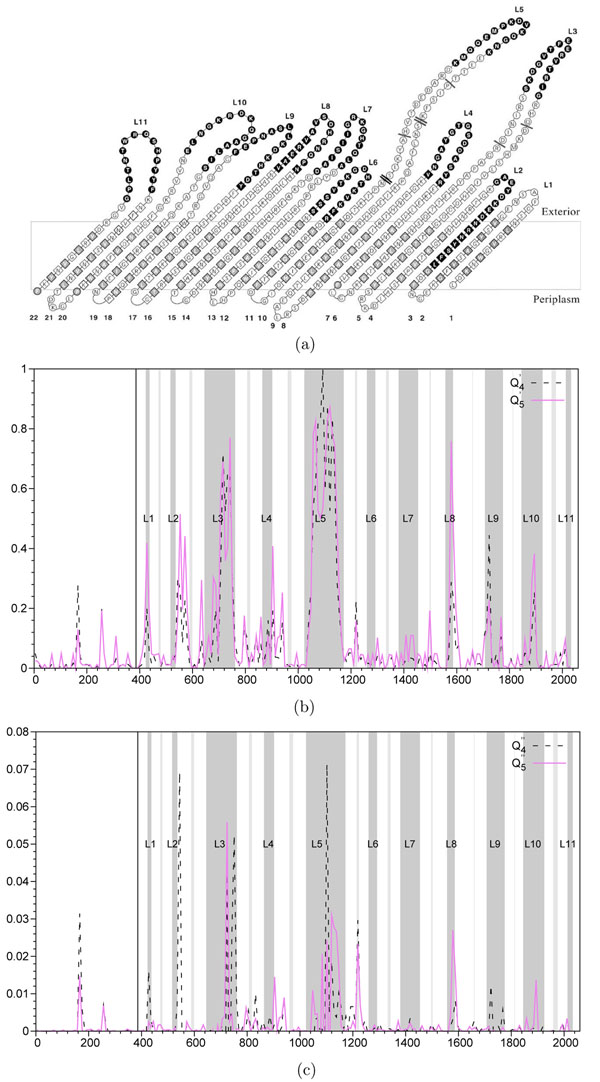
**N. meningitidis FrpB protein variability zone detection** (a) Topology model of the FrpB protein of *N. meningitidis* strain H44/76. Topology of the *β*-barrel. Surface-exposed loops (L) and *β*-strands are numbered. Residues are framed according to their predicted secondary structure: Amino acid residues in *β*-strands are depicted by diamonds. Amino acid residues present in exposed loops and periplasmic turns are depicted by circles (reproduced from Kortekaas et al., 2007) [20]. (b)-(c) Variability zone detection by the hit region identification *Q*′*-tγpe* functions, achieved *without prior knowledge* of invasive taxa (case b), and *Q*″-type functions, *using this prior knowledge* along with the ARI coefficient (case c). Functions  and  are depicted by a dashed line and functions  and  are depicted by a continuous line. A non-overlapping sliding window of size 9 nucleotides was used during the scan of the gene FrpB MSA. The abscissa axis represents the window position along the nucleotide MSA. 11 gray zones correspond to extracellular loops. Annotations start at the solid vertical line (near the 400 abscissa mark).

We scanned the MSA of the FrpB gene using the new algorithm with a sliding window of size 9 nucleotides. We compared the obtained results to the putative topology model of the FrpB protein described in [20] (see Figure 4a). The results are presented in Figure 4b and c.

Remarkably, all surface exposed loops confirmed by enzyme-linked immunosorbent assay (i.e., L2, L3, L4, L5 and L10) [20] were properly detected using the functions  and . It is worth noting that our algorithm was able to find the loop L4, which is hidden between the loops L5 and L3. The model loops L1, L8 and L9 were found at their predicted positions. The loops L2 and L11 were found at different positions, while the loops L6 and L7 were missed regardless of the availability of the prior knowledge information (see Figure 4b and c). As protein models gradually improve and more crystallographic data become available, it will be interesting to reassess these results in the future. Both presented *Q*′-type functions (Equations 5-6) overlap along the alignment, with the exception of the largest loop (L5) and the second largest loop (L3), where the amino acid variability is largely confined. The function  correlates best with surface exposed loops structure. This suggests that the divergences in shape between the functions  and  might be used to detect immunologic activity. It is known that bactericidal antibodies are directed against variable regions situated in the largest loops of proteins [40]. Note that the organisms compared here were strains of the same bacterium; their genetic variant being alleles and evolutionary distances between them being very small. On such a small timescale, underlining evolutionary processes are usually not very diverse. It would be also interesting to verify whether similar conclusions could be made for other outer membrane proteins.

### Human Papilloma Virus analysis

**Figure 5:**
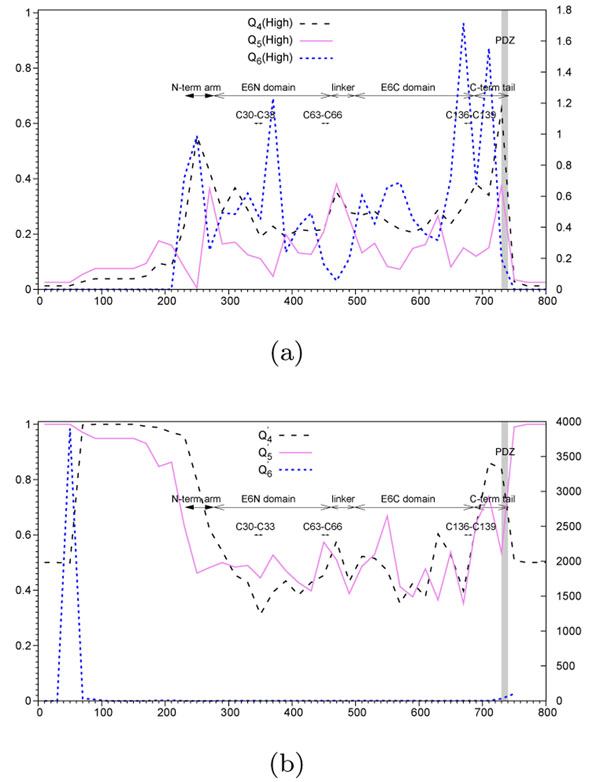
**Hit region identification functions for High-Risk HPV** (a) Functions obtained *using prior knowledge* on the taxa carcinogenicity. The hit region identification functions *Q*_4_, depicted by a dashed line, *Q*_5_, depicted by a continuous line, and *Q*_6_, depicted by a dotted line, for the High-Risk HPV (HPV-16 and 18) [11,12], during the scan of the gene E6. (b) Functions computed *without prior knowledge* on the taxa carcinogenicity. The hit region identification functions *Q'*_4_, depicted by a dashed line, *Q'*_5_, depicted by a continuous line, and *Q'*_6_, depicted by a dotted line, during the scan of the gene E6. The abscissa axis represents the window position along the nucleotide multiple sequence alignment. The *PDZ-doirmm* is highlighted in gray. Annotations for the N and C-terminal arms, E6N and E6C domains are represented for HPV16 coordinates, from (Nominé et al., 2006) [30]. Zn^2+^-ligating Cys residues annotations reproduced from Lipari et al., 2001 [28].

**Figure 6:**
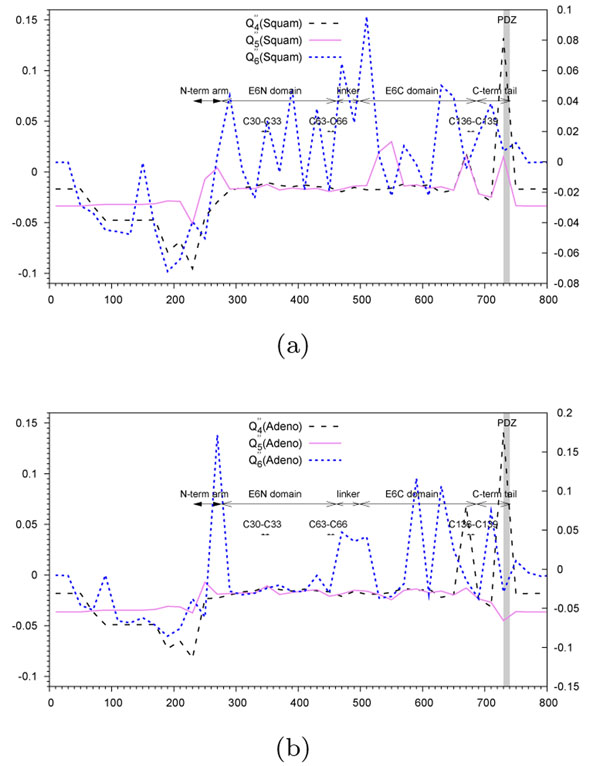
***Q*″-type functions, depending on ARI** (a) Squam HPV dataset. (b) Adeno HPV dataset. Variation of the function *Q"*_4_, depicted by a dashed line, *Q"*_5_, depicted by a continuous line, and *Q"*_6_, depicted by a dotted line, obtained with the non-overlapping sliding window of width 20 nucleotides during the scan of the gene E6. The abscissa axis represents the window position along the nucleotide MSA. The *PDZ*-domain is highlighted in gray. Annotations for the N and C-terminal arms, E6N and E6C domains are represented for HPV16 coordinates, from (Nominé et al., 2006) [30]. Zn^2+^-ligating Cys residues annotations reproduced from Lipari et al., 2001 [28].

We performed a scan of the MSA of the gene E6 for 83 HPV organisms (using non-overlapping windows of size 20 nucleotides). Each time the species bipartition was known, High-Risk HPV against all other HPV types in Figure 5a, Squam-Risk HPV against all other HPV types in Figure 6a, and Adeno-Risk HPV against all other HPV types in Figure 6b, it was incorporated in the computational procedure as shown in Algorithm 1. The comparative results for the High Risk HPV subset provided by the new algorithm *without prior knowledge of carcinogenic taxa* and those yielded by the former one [11], are presented in Figure 5 using annotations for HPV-16. Figure 5a illustrates the results obtained using the functions *Q*_4_ and *Q*_5_* using a prior knowledge* on the species carcinogenicity.

According to the new algorithm, see Figure 5b, the PDZ domain is ranked first in the annotated part of the alignment. A detailed view of the terminal aligned region, within the index interval 680-740, shows a small left shift in the peak positions of the function , but inside the same C-terminal tail domain. On the left side, flanking the PDZ domain, one can find the E6C domain which is related to the DNA binding [30]. One can notice that the function peaks (see Figure 5a and 5b) of  are almost in the same positions than those found using *Q*_4_, exception being a region at the beginning of the alignment (i.e., at the beginning of the E6N domain). As for *N. meningitidis* loops, it would be interesting to study in greater details the regions recognized by both tested functions,  and .

We can conclude, by comparing Figures 5a and 5b, that the new functions,  and , provide almost identical hit region recovery than the existing functions *Q*_4_ and *Q*_5_, which take advantage of a prior knowledge on the species carcinogenicity.

The *Q*″ function validation was also carried out for HPV data. The results are presented in Figure 6. Here, the PDZ domain ranks first for both tested datasets, related to the Squam and Adeno cancers (Figures 6a and 6b). The peaks were found at almost the same positions as in Figure 5, with the exception that only some of the peaks shown in Figure 5 are present here. The function  seems to be less variable than the function . For the Squam dataset, there is one peak in the E6C domain, absent in the Adeno dataset, with a high monophyletic signal and unknown annotation.

On the other hand, the peak located at the index 660, and corresponding to the window positions 660-680, includes two putative Zn^2+^-ligating Cys residues whose absence in mutants results in a dramatic loss in the p53 degradation activity [28].

By analyzing Figures 4, 5 and 6, one can notice that in some situations prior knowledge information brings an important advantage to the method (see the case of Figure 4c when the use of the prior knowledge along with the ARI coefficient allows for getting rid of some false positive hits; for instance, the false positive picks found using *Q*′*-tγpe* functions around the indices 1225 and 1500 presented in Figure 4b were not found by the *Q*″-type functions presented in Figure 4c as well as the case of an almost perfect PDZ domain recovery provided by the *Q*″-type functions as shown in Figures 6a and 6b), but in the other cases, the new algorithm is capable of correct recovering hit regions without any prior knowledge (e.g., see the cases of the loops L1, L3, L5, L8, L9 and L10 for the *N. meningitidis* dataset).

## Conclusion

We described a new algorithm for finding genomic regions that may be associated with a disease. It is capable of detecting hit regions without prior knowledge on the carcinogenicity or invasivity of related organisms. This is an important improvement over previous works in the field [11-13]. We also showed as the Adjusted Rand Index [14,34,35] can be incorporated in the hit detection procedure. The discussed algorithm can be directly used to study organisms that have an ambivalent behavior and are, thus, more difficult to classify. For instance, some strains of *Neisseria Meningitidis* show a hyperinvasive behavior during epidemics, but are non-invasive, otherwise. The behavior of some other organisms, like human papilloma viruses (HPV), is more consistent. Such organisms can be clearly classified with respect to their level of carcinogenicity. Species bipartitions, established according to a carcinogenicity or invasivity criterion, suggested in the literature are important for the identification of genomic regions responsible for a related disease. We showed, however, that a successful identification of these regions can be accomplished without any prior knowledge of the species classification (Figure 5). Considering, in parallel, several hit region identification functions can provide more insight into the structure of genomic regions (Figures 4, 5 and 6). Simulation results suggest that there is no a unique function that provides the best overall results in all practical situations (e.g., the case of monophyletic or polyphyletic evolution and positive or negative selection), and that at least three different functions might be useful (Figures 2 and 3). It is worth noting that the monophyletic scenarios are easier to detect than the polyphyletic ones. The function *Q*_5_ allows for a better detection of monophyletic scenarios, while in the polyphyletic case, the functions *Q*_4_ and *Q*_6_ provide the best results in the positive selection context and in the lineage specific selection context, respectively. The application of the described functions to the HPV gene E6 allows one to retrace the hit regions that are well-known to be related to carcinogenicity [26-28,30].

Furthermore, the results given by these functions while analyzing the FetA sequences of *Neisseria meningitidis* suggest a large overlap between the regions with surface-exposed loops and those detected by the hit region identification functions (Figure 4). All these results indicate the ability of the proposed algorithm to identify regions with bipartite evolutionary signatures according to different patterns of evolution. Each time the species bipartition was known, High-Risk HPV against all other HPV types in Figure 5a, Squam-Risk HPV against all other HPV types in Figure 6a, and Adeno-Risk HPV against all other HPV types in Figure 6b, it was incorporated in the computational procedure as shown in Algorithm 1. In the future, it will be important to assess the correlation between different non-overlapping detected hit regions present in the given alignment. It would be also interesting to compare the performance of the introduced bi-clustering algorithm with the existing bi-clustering methods currently used in bioinformatics, including SAMBA [41], Crossing Minimization [42] and cMonkey [43]. Another possibility consists of using a *k*-means [44] type of algorithms that can suggest partitioning of the given dataset in several, and not necessarily in two, classes when the exact number of classes is unknown. For instance, in the case of HPV data, one could consider the three following HPV classes: High-Risk HPV (types 16 and 18), Low-Risk HPV (types 6, 11, 26, 31, 33, 35, 39, 45, 51, 52, 53, 55, 56, 58, 59, 66, 73, 81, 82 and 83) and No-Risk HPV (all other HPV types).

It is worth noting that the presented algorithm, like most of the comparative genomics methods, relies on the assumption of the alignment correctness. Thus, it will be also important to analyze the impact of alignment errors on the results of the proposed hit detection procedure.

## Competing interests

The authors declare that they have no competing interests.

## Supplementary Material

Additional file 1Algorithm 1. Algorithm for computing genomic regions responsible for carcinogenicity or invasivity.**p-values obtained for hit region detection using the remaining (i.e., not presented in Figs 2 and 3) *Q*′-type functions** (a),(b),(c),(d) Monophyletic evolution - (e),(f),(g),(h) Polyphyletic evolution (a),(c),(e),(g) Positive selection - Variable hit region inside conserved context. Quartile distribution of p-values obtained for the functions , , , and . Abscissa represents scaling factor of the conserved context in which the variable hit region resides. Values close to 0 represent conservation (maximum discrimination), while values close to 1 represent variability (identical to context). Variable hit region is always maintained at a scaling factor of 1. Ordinate represents p-values in log-scale. Horizontal dashed line represents the significance threshold of 0.05. (b),(d),(f),(h) Lineage specific selection - Heterogeneous hit region inside neutral context. Quartile distribution of p-values obtained for the functions , , , and . Abscissa represents the difference in scaling factors among the two lineages present in the hit region. Values close to 0 represent homogeneous evolutionnary speed (similar to the neutral context in which it resides), while values close to 1 represent divergence among these lineages. Context is always maintained at a scaling factor of 0.5, simulating neutral evolution. Horizontal dashed line represents the significance threshold of 0.05. In the case of lineage specific selection, the value of the *Q*′-type functions corresponding to 1 on the abscissa scale cannot be computed because it involves a sub-tree with 0 edge lengths.Click here for file
