# Supplementary material for: Detecting genomic regions associated with a disease using variability functions and Adjusted Rand Index
Source: BMC Bioinformatics. 2011 Oct 5;12(Suppl 9):S9. doi: 10.1186/1471-2105-12-S9-S9 (PMC3271671; doi:10.1186/1471-2105-12-S9-S9)
Supplement: Additional file 1 — Algorithm 1. Algorithm for computing genomic regions responsible for carcinogenicity or invasivity. p-values obtained for hit region detection using the remaining (i.e., not presented in Figs 2 and 3) Q′-type functions (a),(b),(c),(d) Monophyletic evolution - (e),(f),(g),(h) Polyphyletic evolution (a),(c),(e),(g) Positive selection - Variable hit region inside conserved context. Quartile distribution of p-values obtained for the functions , , , and . Abscissa represents scaling factor of the conserved context in which the variable hit region resides. Values close to 0 represent conservation (maximum discrimination), while values close to 1 represent variability (identical to context). Variable hit region is always maintained at a scaling factor of 1. Ordinate represents p-values in log-scale. Horizontal dashed line represents the significance threshold of 0.05. (b),(d),(f),(h) Lineage specific selection - Heterogeneous hit region inside neutral context. Quartile distribution of p-values obtained for the functions , , , and . Abscissa represents the difference in scaling factors among the two lineages present in the hit region. Values close to 0 represent homogeneous evolutionnary speed (similar to the neutral context in which it resides), while values close to 1 represent divergence among these lineages. Context is always maintained at a scaling factor of 0.5, simulating neutral evolution. Horizontal dashed line represents the significance threshold of 0.05. In the case of lineage specific selection, the value of the Q′-type functions corresponding to 1 on the abscissa scale cannot be computed because it involves a sub-tree with 0 edge lengths. [file 1471-2105-12-S9-S9-S1.pdf]

Algorithm 1

Algorithm 1. Algorithm for computing genomic regions responsible for carcinogenicity or invasivity

Require: *FI*: Hit region identification function to be optimized Q_4, Q_5 or Q_6,
MSA: Multiple sequence alignment,
X: Subset of carcinogenic or invasive taxa,
Y: Subset of non-carcinogenic or non-invasive taxa,
WIN_MIN: Minimum sliding window width,
WIN_MAX: Maximum sliding window width,
S: Sliding window step,
RPG: Constant number of random bipartition generations.

Ensure: Set of Hit Regions: (win_width, idx, Q', ARI, Q''), where
win_width : Current sliding window width,
idx : Hit Index (i.e., its genomic position),
Q' : Hit region identification function without knowledge of X and Y,
ARI : Adjusted Rand index,
Q'' : Validation function depending on ARI.

$MSA\_L \leftarrow$ Length of MSA
for win_width **from** WIN_MIN **to** WIN_MAX **do**
  for idx **from** 0 **to** $MSA\_L - win\_width$ **with step** S **do**
    for all r such that $1 \leq r \leq RPG$ **do**
      Randomly select a bipartition $A|B$
      $\mathrm{MSA_A} \leftarrow \mathrm{MSA}[A][idx..idx + win\_width]$
      $\mathrm{MSA_B} \leftarrow \mathrm{MSA}[B][idx..idx + win\_width]$
      $Q_{Partition}$ = Calculate $Q(FI$, A, B, $\mathrm{MSA_A}$, $\mathrm{MSA_B})$
      Update $Q' = Max(Q_{Partition}, r)$
      repeat
        for all $i \in A$ **do** // i is randomly chosen
          $A \leftarrow A \setminus i$, $B \leftarrow B \cup i$
          Update $Q_{Partition}$
          $MaxQ_{Partition} = Max(MaxQ_{Partition}, Q_{Partition}, r)$
          keep old A and B if $MaxQ_{Partition}$ is unchanged
        end for
        Swap (A,B)
      until No improvement of $MaxQ_{Partition}$ is possible
      $Q'[win\_width, idx] = Max(Q', MaxQ_{Partition})$
      $ARI[win\_width, idx]$ = Calculate ARI($A|B$, $X|Y$)
      $Q''[win\_width, idx] = ARI \times Q'$
    end for
  end for
end for
return Q', ARI, Q''

Additional file 1 (a), (b) - Remaining monophyletic evolution hit detection p-values

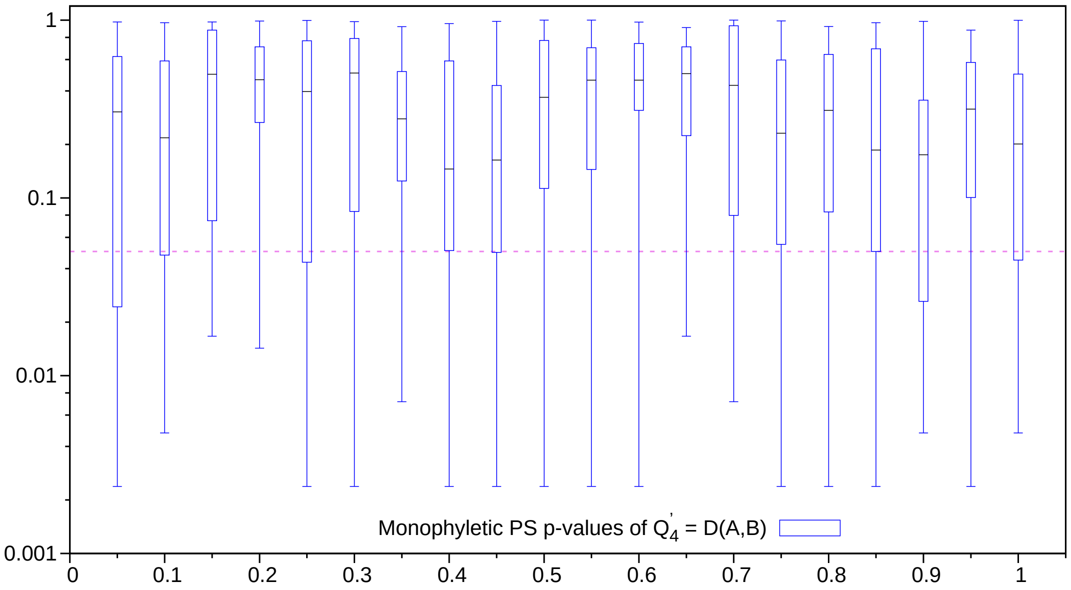

(a)

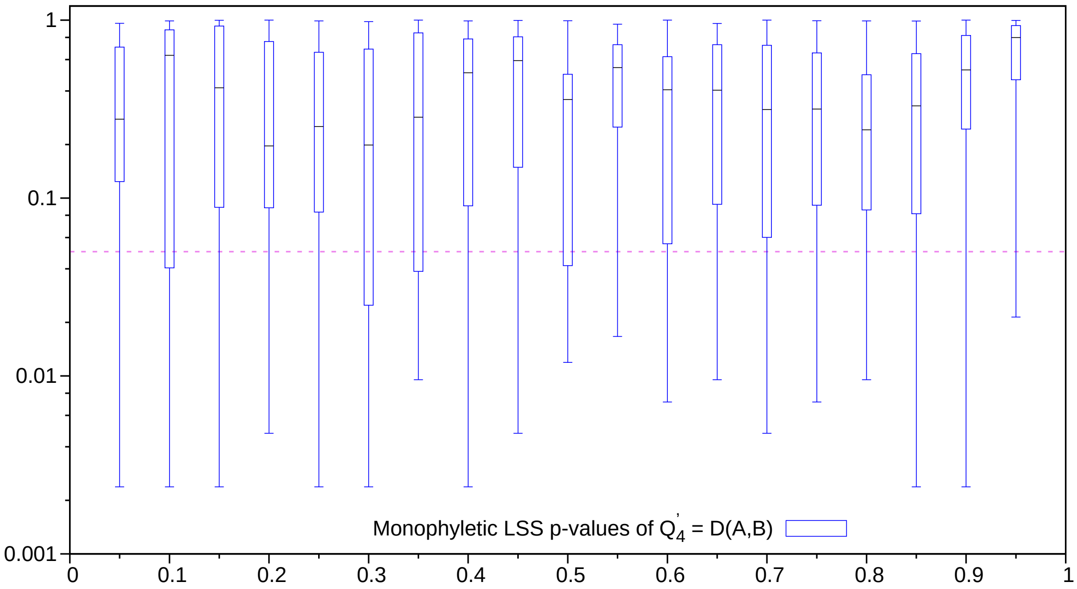

(b)

Additional file 1 (c), (d) - Remaining monophyletic evolution hit detection p-values

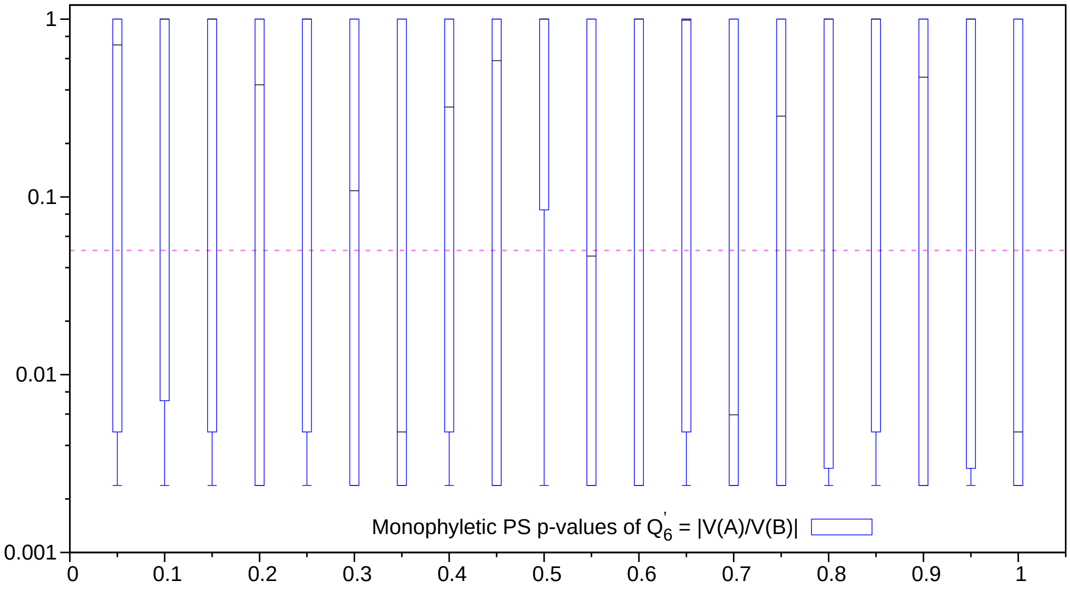

(c)

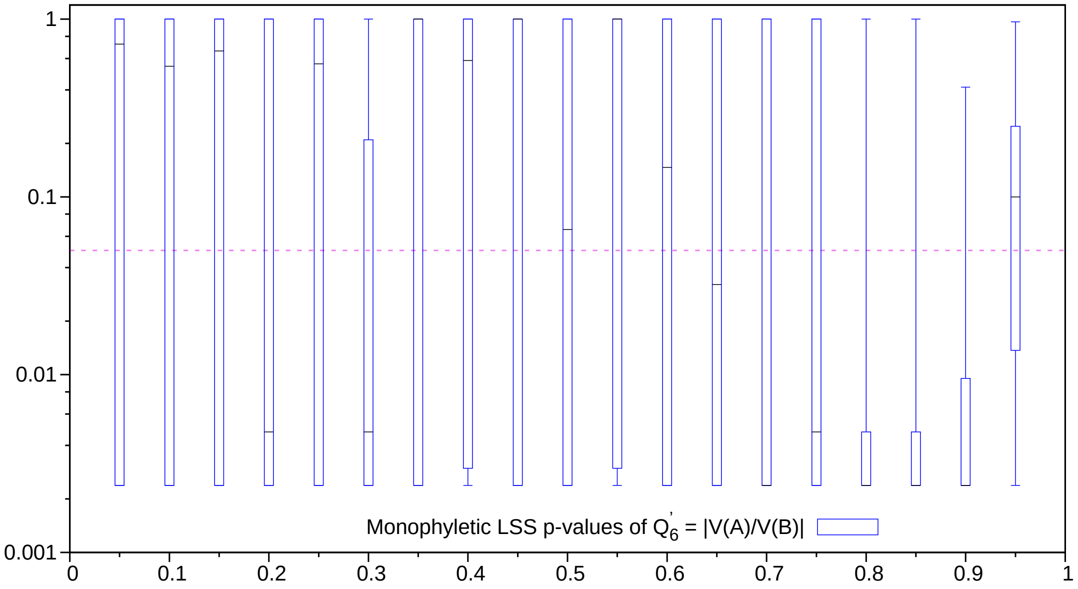

(d)

Additional file 1 (e), (f) - Remaining polyphyletic evolution hit detection p-values

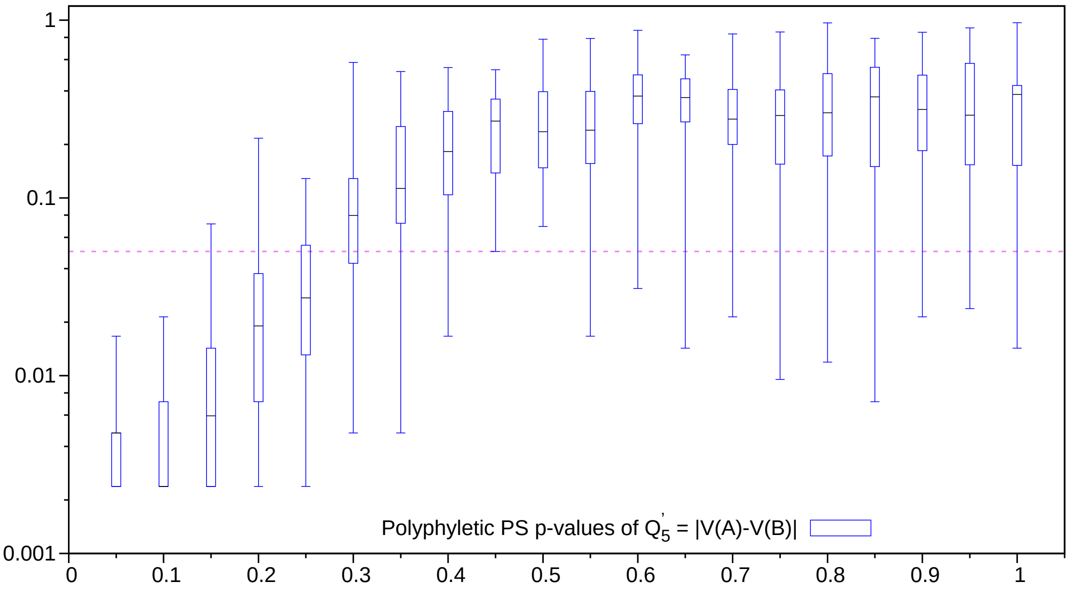

(e)

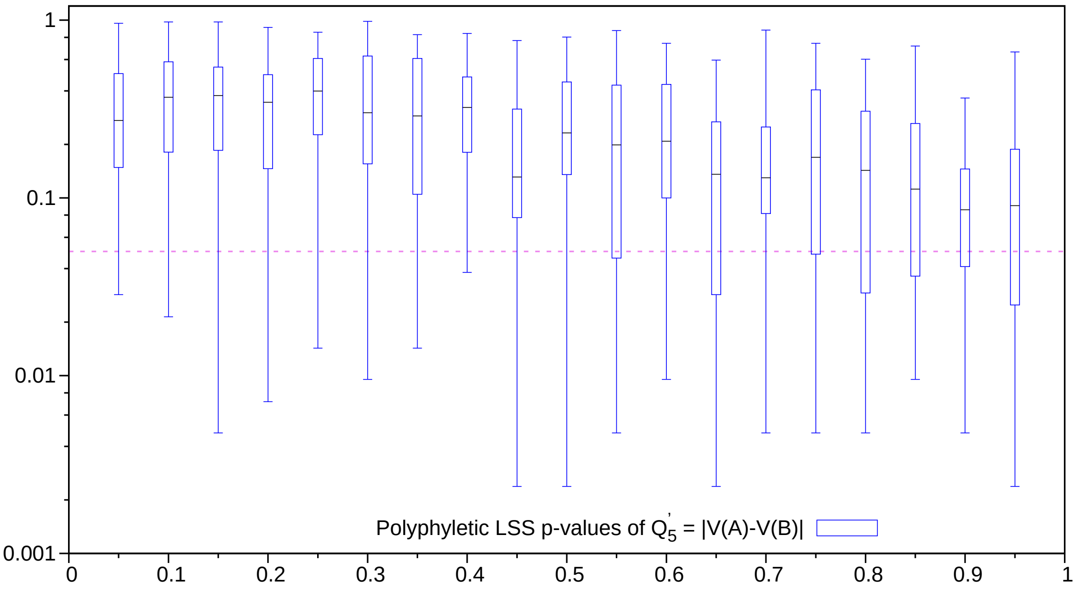

(f)

Additional file 1 (g), (h) - Remaining polyphyletic evolution hit detection p-values

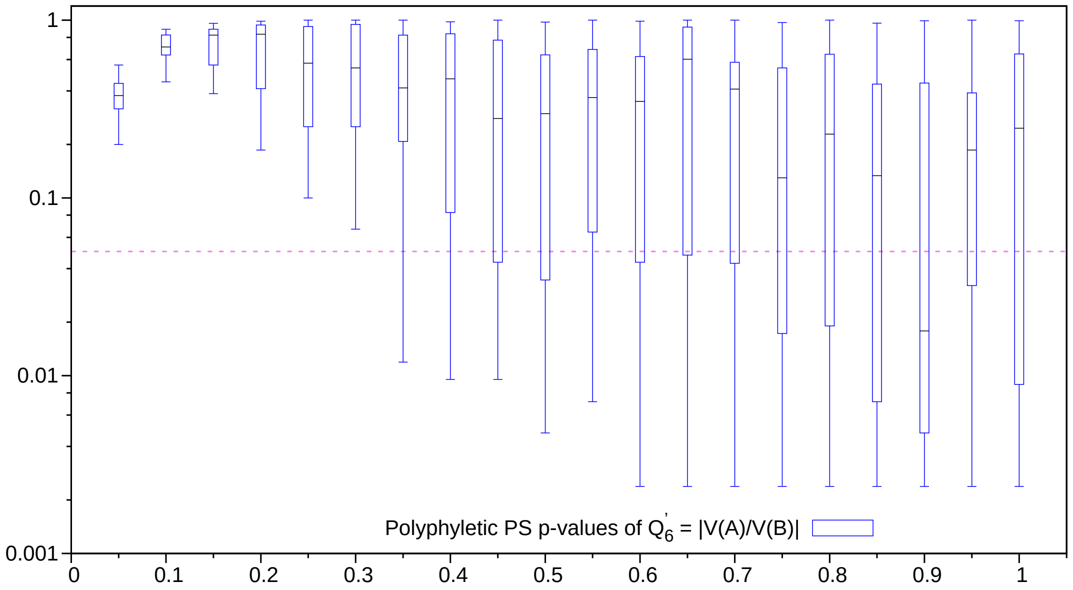

(g)

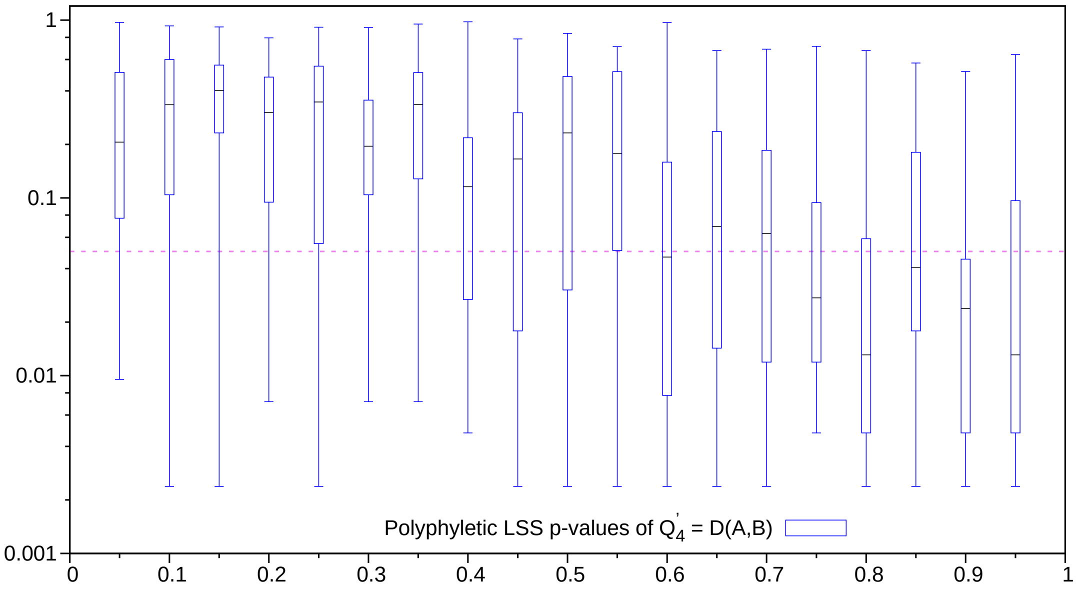

(h)
